# The Ecological Associations of Surface-Dwelling Lizards in Qom Province in the Northwest of Central Plateau of Iran

**DOI:** 10.1371/journal.pone.0083890

**Published:** 2013-12-13

**Authors:** Mehregan Ebrahimi, Faraham Ahmadzadeh, Hossein Mostafavi, Aahmad Reza Mehrabian, Asghar Abdoli, Abdolrasoul Salman Mahini

**Affiliations:** 1 School of Biological Sciences, Flinders University, Adelaide, South Australia, Australia; 2 Department of Biodiversity and Ecosystem Management, Environmental Sciences Research Institute (ESRI), Shehid Beheshti University, Tehran, Iran; 3 Zoologisches Forschungsmuseum Alexander Koenig, Adenauerallee, Bonn, Germany; 4 Department of Botany, Faculty of Biological Sciences, Shahid Beheshti University, Tehran, Iran; 5 Department of the Environmental Sciences, Gorgan University of Agricultural Sciences and Natural Resources, Gorgan, Iran; State Natural History Museum, Germany

## Abstract

We used pitfall trapping to investigate the effects of elevation, plant density and soil structure on species diversity and the impact of these habitat factors on lizard habitat selectivity in the Qom Province in the Central Plateau of Iran. From a total of 12 1-ha plots, we captured 363 individuals of 15 species of lizards (six species of Lacertidae, five species of Agamidae, two species of Gekkonidae, one species of Varanidae and one species of Scincidae). A generalized linear model (GLM) determined that elevation was the most important factor impacting species diversity. The highest species diversity was at the intermediate elevation (1289 m). Abundance of 6 out of 15 species showed strong relationships with some habitat factors. These relationships were demonstrated by habitat selectivity index (Ivlev's index). Our result supports other surveys that showed that elevation plays an important role in determining lizard species diversity.

## Introduction

Ecologists have investigated patterns of species diversity and the mechanisms of community assembly for many years [1], and lizards have often been used as model systems to explain patterns of species diversity [2-4]. Lizards show extensive inter- and intraspecific variation in patterns of space use [3,5] and distribution, depending on environmental factors such as elevation, soil structure, vegetation [6,7] , as well as temperature and precipitation, which are usually correlated with elevation. These physical properties of the environment have an important role in defining the relationship between habitat and lizard species diversity. For instance Shenbrot and Krasnov [8] classified habitat types using factors that described vegetation and soil structure. Then they reported that lizard richness, diversity and biomass changed among these habitat types within different landscapes. For instance they showed *Mesalina olivieri* avoids habitat with soil structure that have moderate gravel content. 

The species richness (actual number of species), diversity (effective number of species, which takes into account both richness and evenness) [9,10], and biomass of lizards can vary among habitats, because each species responds differently to abiotic and biotic factors. Variation in topography provides transitions across multiple habitats on a regional scale (mesoscale), which may show the role of elevation in community structure [11]. Species diversity, across all taxa, generally decreases with increasing elevation [7], but this decline is not necessarily linear, which has been shown in some species of birds and insects [12]. Amphibians and reptiles follow this general pattern and their species diversity decreases with increasing elevation [13]. 

More recent studies have shown that there could be three other patterns of reptile species diversity. All three patterns demonstrate a generally decreasing species diversity with increasing elevation but with a diversity peak at low plateau, mid-elevation or at both [14]. McCain [14] also showed that ambient temperature is strongly correlated with these patterns (low plateau, mid-elevation and low plateau with mid-elevation), in particular along wet mountain gradients. In these areas opportunities to use radiant heat for thermoregulation are limited. On the other hand, in arid environments the effect of ambient temperature is reduced because radiant heat is not limited, however ambient temperature is influenced by vegetation density, soil texture and rainfall [14]. 

In this study, we examined lizard species diversity and its association with major habitat features such as elevation, vegetation and soil structure through pitfall trapping in Qom Province in Central Plateau of Iran which is considered as an arid environment. Species from the Palearctic, Oriental, and Afrotropical provenances [15,16] are found in this part of the Central Plateau of Iran. However, little is known about the lizard biodiversity in this region. We address three questions in this paper. 1) Does elevation influence lizard species diversity? 2) Are there additional habitat factors that play an important role in species diversity? 3) What is the effect of habitat factors on individual species abundances and how strong is their effect? 

## Materials and Methods

### Study area

**Figure 1:**
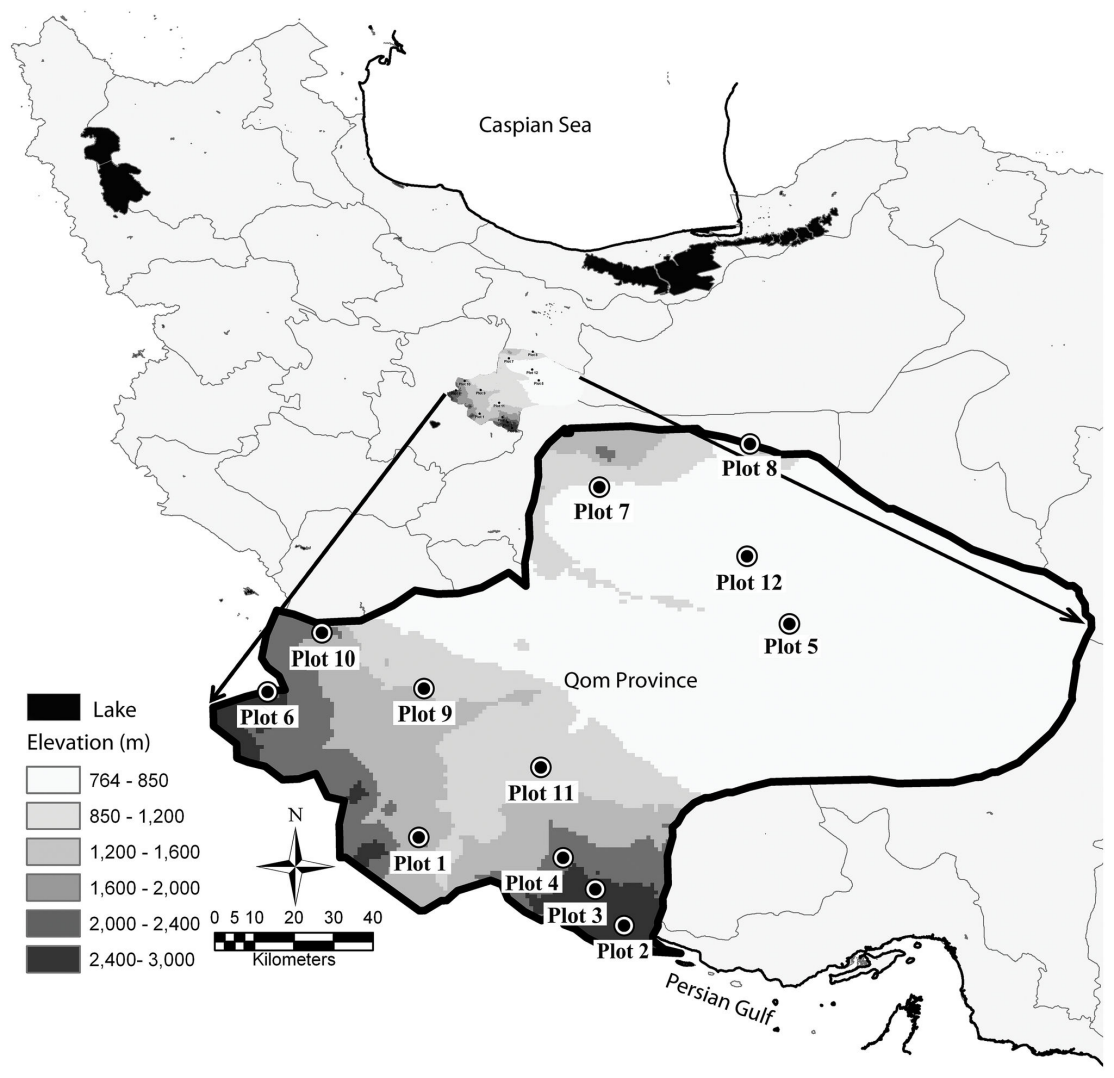
The plots distribution in the Qom Province of Iran. The plot numbers correspond to those in Table 1.

The Qom Province (34° 08’ to 35° 09’N, 50°08’ to 52°08’E, datum = WGS 1984) forms the Northwest of the Iranian Central Plateau. It expands over a total area of approximately 11,238 km^2^ and ranges in elevation from 600 to 3300 m. It has hot summers and cold winters; average monthly temperatures in this area range from 15.2 to 40.1°C during summer and –1.7 to 18°C during winter. The average annual precipitation is 150 mm (range = 0.5 - 28.1 mm / month). The Qom Province ranges from a mountainous landscape in the south and west which includes the Zagros Mountain to a desert landscape in Central Kavir in the east (Figure 1). 

### Ethics

The research was carried out under scientific permits from the Iranian Department of Environment and adhered to the animal welfare regulations in Iran. Under these regulations a particular animal welfare license was not required, because we did not remove, toe clip or sacrifice animals. All caught lizards were released at the point of capture after identifying the species.

### Lizards and data collection

We conducted our study from June–August, 2006, in 12 one ha plots. The plots were chosen such that they represented the main elevation and vegetation gradients [8,17]. We used satellite maps and field work to determine the elevation and vegetation gradients and to assign plot locations (Figure 1). The field work included identifying agricultural lands, checking areas that were ambiguous in the map and gathering general information of these areas in order to assist with selecting the plot locations. In our analyses we divided elevation into three categories, low (less than 1100 m, five plots out of 12), intermediate (between 1100 and 1800 m, three plots), and high elevation (above 1800 m, four plots). The measured elevations of each plot are given in Table 1. Our sampling method involved pitfall trapping. We placed 75 pitfalls (50 small and 25 large) in each one hectare plot. Each small trap had a 62.5 cm depth and 10 cm diameter and each large trap had depth of 62.5 cm and 20 cm diameter. To distribute pitfall traps equally across the one hectare we divided each plot into 25 subplots of 20 × 20 m each. The traps were placed at random within each subplot while one large and two small traps were allocated to each subplot and were buried into the ground so that their entrances were flush with the ground surface. Pitfalls were checked once every 7 days for 3 months.

**Table 1:** Number of individuals of each lizard species captured in each 1-ha plot (rows) in the Qom Province of Iran, and the species diversity among 12 plots (columns).

	**Plot number**											
**Species**	**P1**	**P2**	**P3**	**P4**	**P5**	**P6**	**P7**	**P8**	**P9**	**P10**	**P11**	**P12**
	**(1452 m)**	**(2942 m)**	**(2390 m)**	**(1931 m)**	**(836 m)**	**(2214 m)**	**(951 m)**	**(1069 m)**	**(1289 m)**	**(1709 m)**	**(1041 m)**	**(831 m)**
*Ophisops elegans*	23	-	1	5	-	3	1	-	8	95	-	-
*Agamura persica*	3	-	-	1	1	-	9	1	-	-	1	1
*Bunopus carssicauda*	6	-	-	-	6	-	-	1	1	1	2	2
*Trapelus agilis*	16	-	-	-	5	-	13	14	4	14	22	-
*Trapelus lessonae*	-	-	-	-	-	1	-	-	-	-	-	-
*Varanus griseus caspius*	-	-	-	-	-	-	-	-	1	-	-	-
*Eumeces schneiderii princeps*	-	-	-	1	-	1	2	4	8	-	25	-
*Laudakia caucasia*	-	-	1	-	-	-	-	-	-	-	-	-
*Phrynocephalus scutellatus*	-	-	-	-	-	-	4	-	-	1	-	-
*Phrynocephalus maculatus*	-	-	-	-	-	-	-	-	-	-	-	8
*Mesalina watsonana*	2	-	-	-	-	-	1	1	-	-	7	-
*Eremias fasciata*	-	-	-	-	18	-	-	-	-	-	-	-
*Eremias velox*	-	-	-	-	1	-	-	-	-	-	-	-
*Eremias persica*	8	-	-	5	-	-	-	-	-	-	1	-
*Eremias andersonii*	-	-	-	-	2	-	-	-	-	-	-	-
Number of specimens captured	58	0	2	12	33	5	30	21	22	111	58	11
Species diversity	3.93	0	2.5	2.7	3.6	3.01	3.5	3.02	4.15	2.47	3.2	2.4
( Hill's N2 index)												

The values for species diversity derived from Hill's N2 index, as described in the text.

Captured lizard species were identified using the field guide “The Lizards of Iran” [16]. Lizards were also sexed, weighed, and marked with a small dot of paint on its dorsal surface, hind and front leg, and tail, and then released [18]. If the lizard was captured only once it had one mark, if it was caught twice it had two marks and so on. 

Vegetation sampling was conducted following the Braun-Blanquet cover scale [19]. The vegetation quadrats were selected based on minimal area. In this method we established a starting point for quadrat sampling. Point selection was based on the homogeneity of the vegetation in the studied quadrat. The quadrat size was stratified to the vegetation type: annual, perennial, and shrub corresponded to quadrat sizes was between 4 and 32 m^2^. We recorded the dominant and companion species and their percent ground cover in each plot. We identified vegetation types for each quadrat based on the presence of perennial species and also accompanying companion species. We named each vegetation type after the most dominant plant species. We then extrapolated the measured percentages of vegetation ground cover of the quadrats to the size of the one hectare plot in order to evaluate the abundance-dominance [19]. We summarized the total area each vegetation class (annual, perennial, shrubs) covered and divided it by the total area surveyed in each plot. We then further divided these relative values into 5 categories of relative ground cover 1 (0–5%), 2 (6–25%), 3 (26–50%), 4 (51–75%) and 5 (76–100%). We conducted vegetation surveys every month from February through October (spring through the end of summer) of 2006. We used overall plant cover, annual, perennial and shrub plant cover for each plot as vegetation variables in our study. 

To determine whether soil structure impacts lizard habitat preference, we took a 0.5 kg core sample of soil from the center of each plot. We then sent these samples to the soil laboratory at the Pishahang institute, Qom Province, Iran. The soil texture was determined using the USDA textural system (clay, sand and silt) [20,21],water absorption was also measured [22]. Each of these soil factors were categorised to three levels, low (0-20%), medium (21-40%) and high (>41%). 

### Data analysis

In our statistical analysis of what drives lizard abundance and lizard diversity we used 10 environmental variables consisting of elevation, 4 vegetation variables and 5 soil structure variables. The dependent variable (abundance and diversity indices) was calculated for each of 12 plots. The total number of each species caught in each plot over the sampling period was then used for identifying species diversity and each species abundance in each plot. We excluded 12 recaptured lizards from the analysis, to avoid pseudo replication. All other individuals were caught only once during the study. 

### Relationship of species diversity and elevation

First we used Hill's N2 index (reciprocal of Simpson's index) to determine species diversity for each plot [23]. We used this index because it is particularly suitable when a single species dominates the diversity in a plot, [24], which is the case in some of our plots. We used the rarefaction method in the EstimateS freeware [25] to achieve equal sample sizes which is an important step prior to calculating the Hill’s N2 index. Then we used regression with quadratic term to address how elevation affects species diversity. We used species diversity (Hill's N2) as dependent factor and elevation as independent factor. 

### Relationship of species diversity (Hill's N2 index) and habitat variables using GLM

We used a generalized linear model (GLM) to examine the effect of elevation, vegetation, and soil structures on species diversity to address which habitat variables affect species diversity. We used the Hill's N2 index as the dependent variable and three elevation categories, five overall plant cover categories and two soil water absorption categories as predictor factors in our final model. In order to identify all significant interactions between habitat factors and species diversity (Hill's N2 index), we used a factorial model. Due to the limited number of degrees of freedom (12 plots were sampled) we could not include all sampled 10 environmental variables into our model. Instead we followed Shenbrot and Krasnov [8] and used Pearson correlations to determine whether variables were included as factors or covariates into the model. Variables that were highly correlated with the dependent variable (Hills N2 index; r>0.7) were included as covariates (annual and shrub plant cover, percent of sand, silt and gravel) [26]. Finally, we calculated different models of GLM based on different combination of predictor factors that we added or excluded from model and selected the model with the lowest value of the Akaike Information Criterion (AIC). This model shows the best possible relationship between habitat variables and species diversity. The model was also ranked by chi-square to show precision of the estimate of our model. 

### Relationship of each species abundance and habitat variables

To address which habitat factors affect individual species abundances we used analysis of deviance assuming Poisson error distribution [27], a method suggested for data with low numbers of caught individuals or with many zeros in the data matrix [28]. We conducted this analysis on 6 species (Table 2) after omitting all species that were only captured in one plot and in low numbers or that were only recorded once. Before we started our analysis we tested all habitat variables against each species abundance separately to find possible covariates as it was described above. Then we analysed each species separately with the species abundance as the dependent variable, habitat variables which were not covariates were independent variables, and habitat variables which were covariates as covariates. This analysis determines which habitat variable is the most important for each lizard species. Finally we used Ivlev's index as a measure of habitat selectivity [8]. This index ranked the preference of each lizard between -1 and 1 for each habitat variable that we identified in the analysis of deviance. For this ranking we needed to divide each habitat variable into intervals. We choose 5 intervals for each of our habitat variables. These habitat intervals include elevation (<1050, 1050-1550, 1551-2050, 2051-2551 and above 2551 m), overall plant density (0%, 1-20%, 20.1-30%, 30.1-40% and 40.1%>), annual plant density (0%, <10%, 10.1-20%, 20.1-30% and 30.1%>), clay (0%, <13%, 13.1-15%, 15.1-17%, and 17.1%>), soil water absorption <23%, 23.1-26%, 26.1-29%, 29.1-32%, 32.1>) and perennial plant density (0%, 50-60%, 60.1-71%, 71.1-81%, 81.1%>). 

**Table 2:** Relationships between habitat features and lizard abundance in the Qom Province of Iran.

**Species**	**Variable**	**Parameter estimate**	**F value**	**df**	**Sig. <0.05**	**AIC**
*Ophisops elegans*	Overall plant cover	0.170	6.53	1	**0.030**	253.00
	Elevation	0.010	1.78	1	0.230	301.00
	*Gravel*	-0.050	0.66	1	0.440	410.00
	*Relative annual plant cover*	0.030	0.57	1	0.470	413.00
*Bunopus crassicaudatus*	*Relative annual plant cover*	-0.090	7.39	1	**0.020**	40.41
	S.W. absorption	-0.180	0.33	1	0.590	44.00
	Elevation	-0.002	3.84	1	0.090	45.00
	*Relative shrub plant cover*	0.050	2.38	1	0.150	48.00
*Trapelus agilis*	S.W. absorption	-0.270	14.04	1	**0.005**	69.88
	Elevation	-0.002	6.76	1	**0.030**	85.75
	Overall plant cover	0.030	0.65	1	0.440	85.70
	*Gravel*	0.030	1.15	1	0.310	137.00
*Eumeces schneiderii princeps*	*Clay*	-0.550	13.90	1	**0.007**	41.93
	S.W. absorption	-0.370	7.93	1	**0.020**	70.89
	Elevation	-0.003	5.84	1	**0.040**	84.00
	*Relative annual plant cover*	0.030	0.64	1	0.440	124.00
*Phrynocephalus scutellatus*	*Relative annual plant cover*	0.100	4.83	1	**0.005**	22.64
	Elevation	-0.002	1.84	1	0.200	26.00
	Overall plant cover	-0.030	0.71	1	0.420	28.00
	*Gravel*	0.001	0.02	1	0.890	29.00
*Mesalina watsonana*	S.W. absorption	-0.680	7.95	1	**0.030**	23.29
	Elevation	-0.004	16.53	1	**0.004**	26.00
	Relative perennial plant cover	0.100	7.49	1	**0.030**	26.00
	Overall plant cover	0.070	4.12	1	0.080	26.00

The models are ranked by AIC values, with the lowest values indicating the most important models. Only the species that had significant associations with habitat variables are shown, S.W. absorption = Soil water absorption; italics indicate that a variable was included as covariate; bold numbers indicate significant associations between variable and the abundance of that lizard species.

All analyses were conducted using R [29] and IBM SPSS Statistics 20. 

## Results

In total, we captured 363 individuals of 15 lizard species in the 12 plots (Table 1). *Ophisops elegans* (37.5% of all captures) and *Trapelus agilis* (24.2%) were the most abundant lizard species in our plots. 

### Relationship of species diversity and elevation

**Figure 2:**
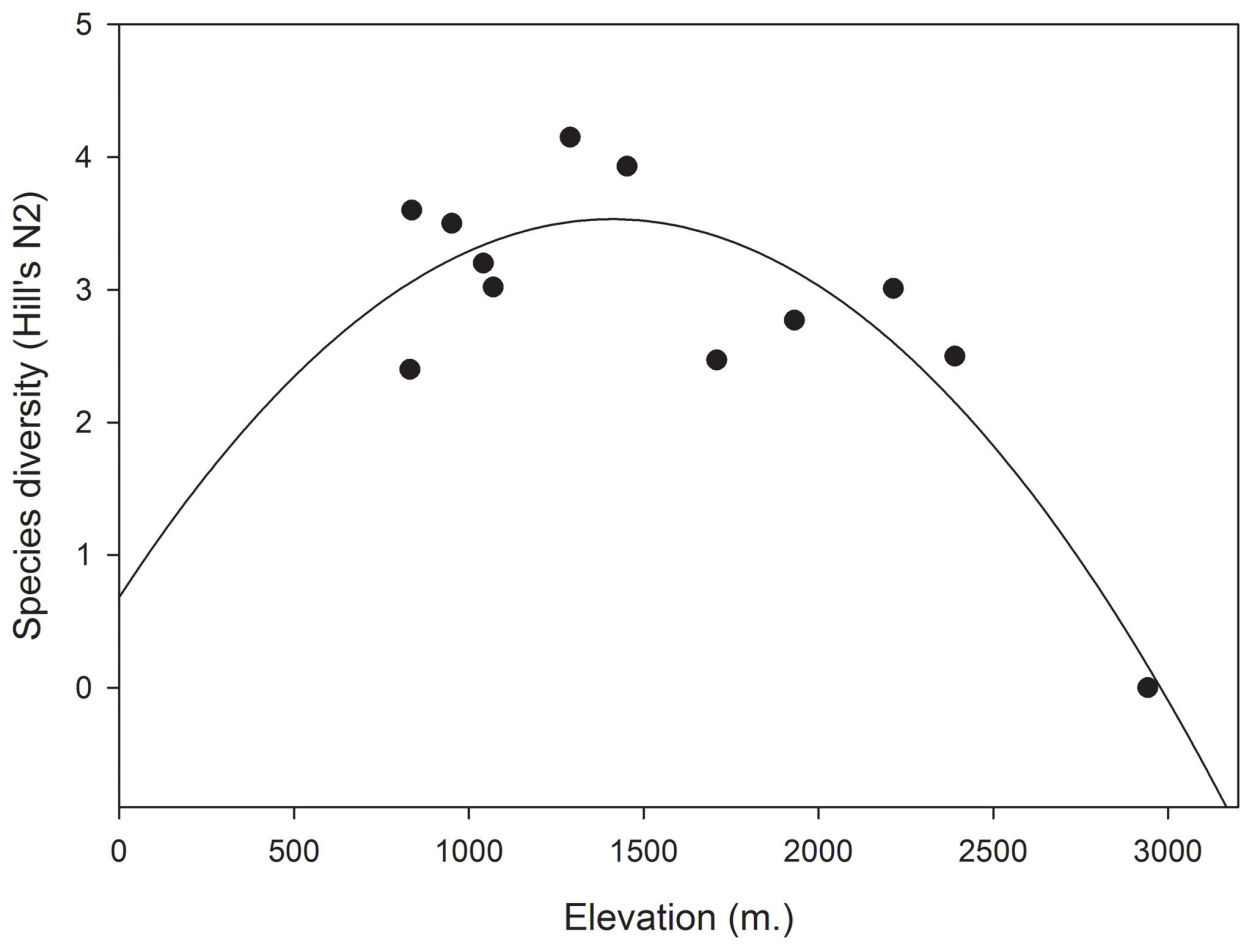
Pattern of lizards species diversity (Hill's N2 index) and elevation in the Qom Province of Iran.

Species diversity (Hill's N2) in each plot ranged from 0 to 4.15 (Table 1). The regression with quadratic term demonstrated that species diversity was highest at intermediate elevation (Table 3: Figure 2).

**Table 3:** Relationship of elevation and species diversity according to regression analysis with quadratic term.

	**Coefficient**	**t**	**p value**	**R^2^**
**a**	0.00400	2.742	0.023	0.770
**B**	-0.00002	-3.514	0.006	

### Relationship of species diversity (Hill's N2 index) and habitat variables using GLM

**Figure 3:**
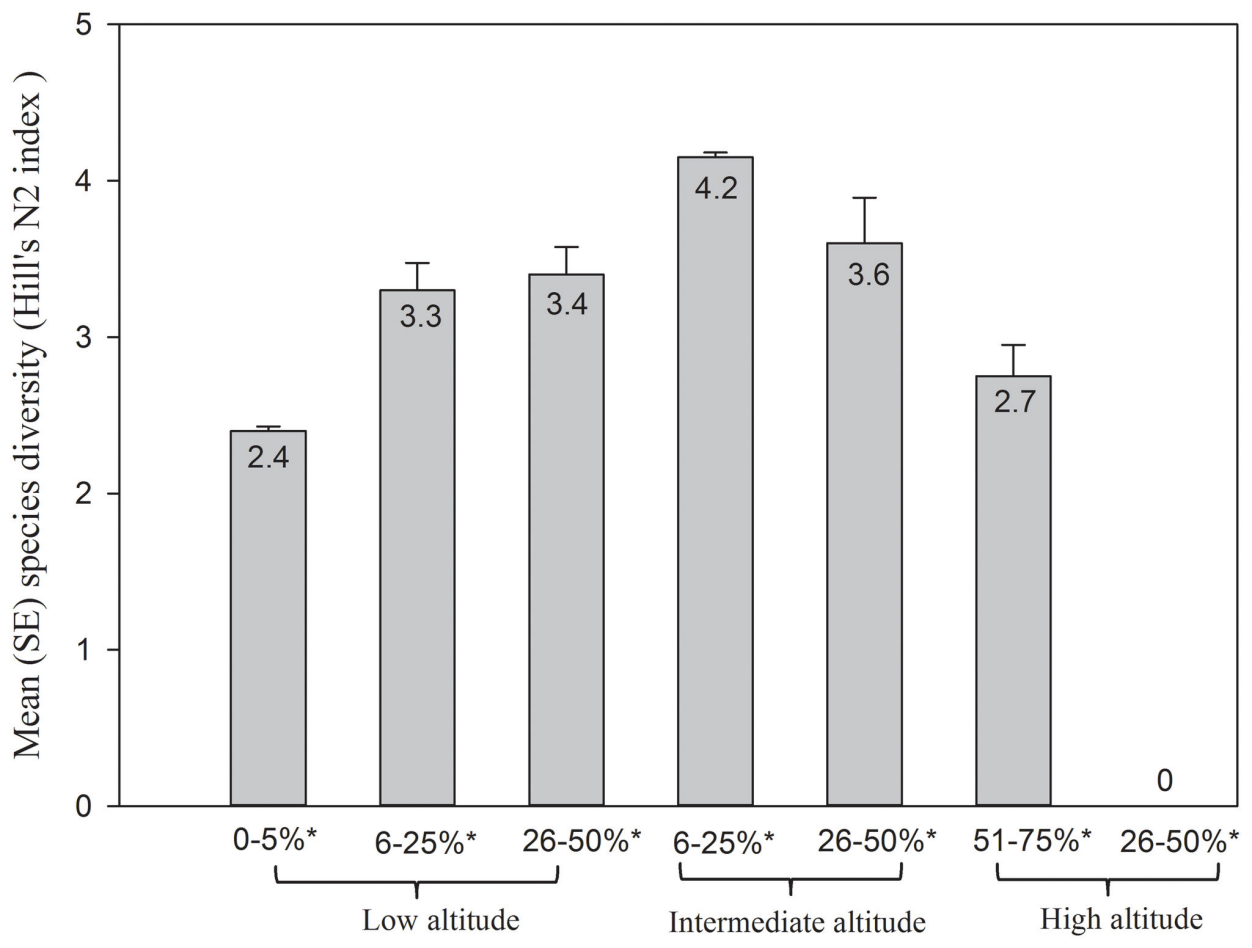
Pattern of lizard species diversity (Hill's N2 index) in relation to overall plant density (percentage with star) and elevation in the Qom Province of Iran. Low elevation <1100 m, medium elevation 1101-1800 and high elevation >1801 m.

**Figure 4:**
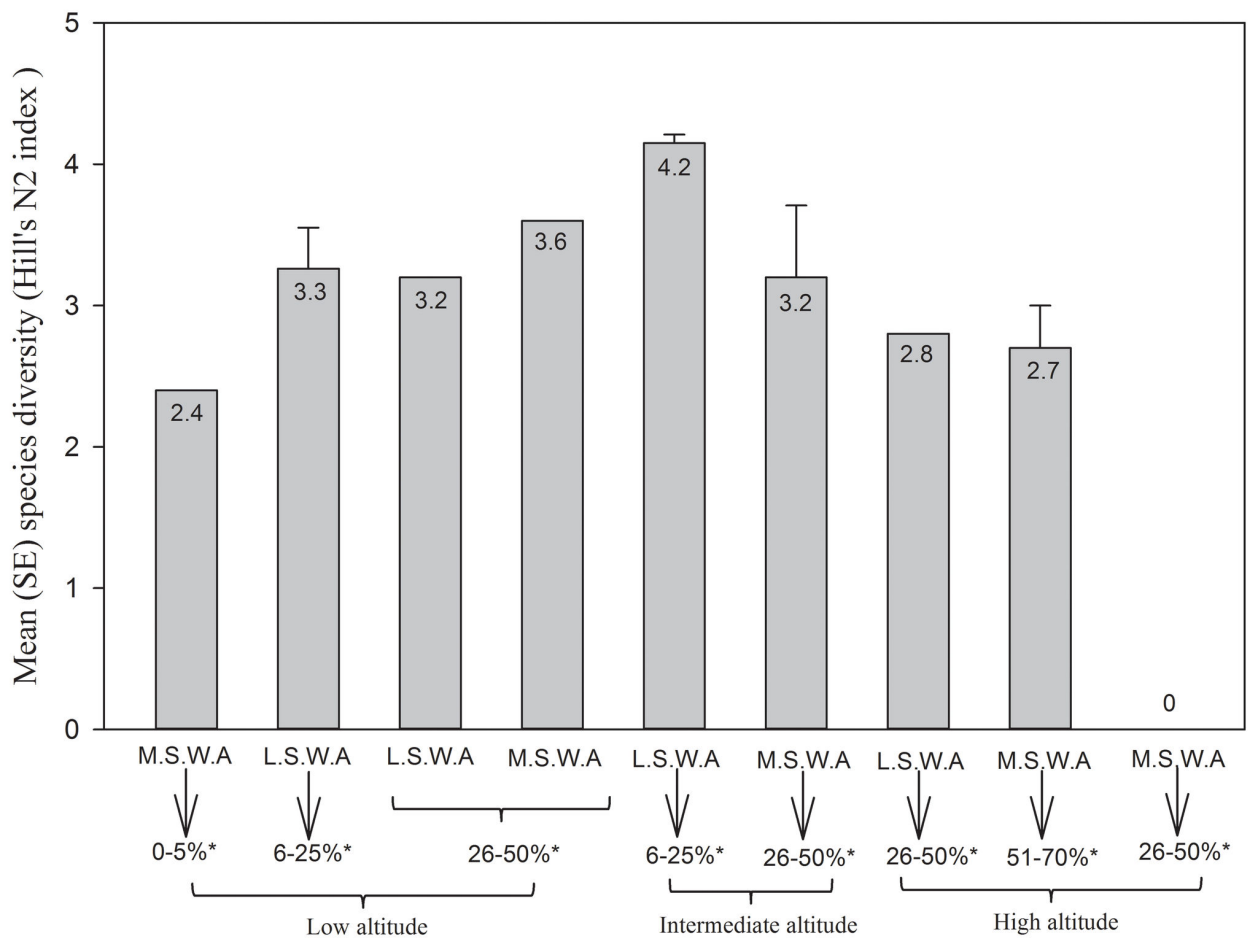
Pattern of lizard species diversity in relation to soil water absorption (S.W.A), overall plant density (percentage with star) and elevation in the Qom Province of Iran. Low soil water absorption (L.S.W.A) <24.1, medium soil water absorption (M.S.W.A) >24.1. Plant density and elevation categories are same as Figure 2.

Species diversity (Hill's N2) in each plot ranged from 0 to 4.15 (Table 1). The generalized linear model also demonstrated that elevation was one of the most important variable associated with species diversity among the 12 plots (Table 4). The following interactions also had a significant influence on the species diversity (Table 4): soil water absorption × elevation, overall plant cover × elevation, and overall plant cover × soil water absorption × elevation. The lizard species diversity increased as overall plant cover and elevation increased; however, species diversity declined as elevation and overall plant cover were still increasing (Figure 3). No lizards were found at elevations exceeding 3000 m (Plot 2). Species diversity increased at low elevations (less than 1500 m), where overall plant cover reached moderate percentages (25%–40%), and decreased at high elevations (above 1500 m.), where overall plant cover rose to high percentages (over 40%). When we included the next interaction (soil water absorption), the trend of species diversity remained the same as Figure 2, but species diversity was highest at low values of soil water absorption (less than 25 %), with the moderate percentage of overall plant cover and low elevation. Species diversity started to decrease when soil water absorption reached a moderate percentage (25%–40%), and decreased from mid to high elevations, from medium to high plant covers, and from low to medium percentages of soil water absorption (Figure 4). 

**Table 4:** Relative importance of habitat variables and their interactions (×) to species diversity according to GLM (generalized linear model) analyses.

**Dependent variable**	**Independent variable**	**Wald Chi-Square**	**df**	***P* value**
Species diversity **(Hill’s N2**)	Elevation	9.11	1	**0.003**
	Overall plant cover × Elevation	6.86	1	**0.009**
	Soil water absorption × Elevation	8.65	1	**0.003**
	Overall plant cover × Soil water absorption × Elevation	5.04	1	**0.030**
	Soil water absorption	3.68	1	0.060
	Overall plant cover	1.16	1	0.280
	Overall plant cover × Soil water absorption	0.35	1	0.560

The factors are ranked by chi-square values.

### Relationship of each species abundance and habitat variables

**Figure 5:**
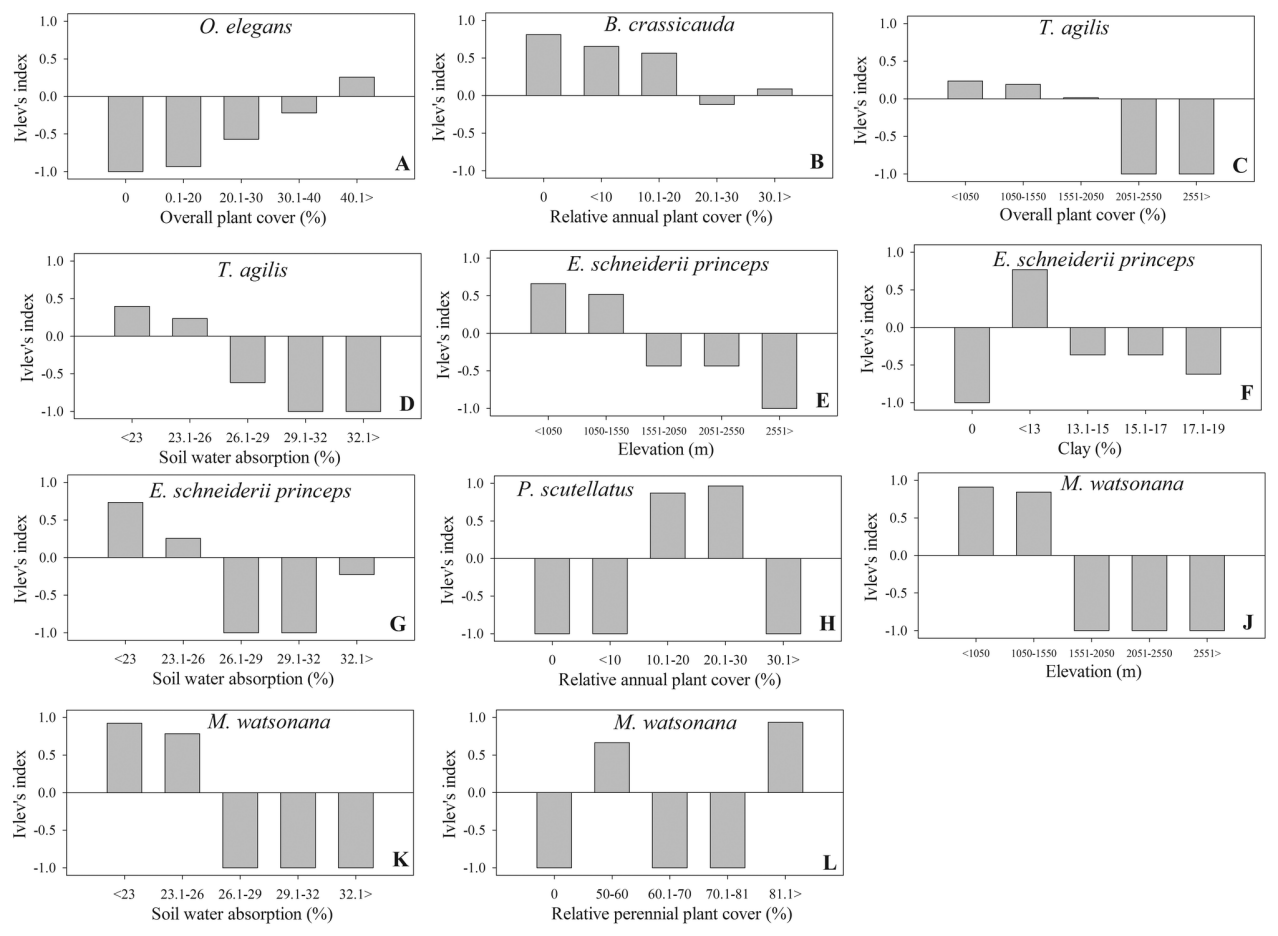
Habitat variable ranks for six lizards species in the Qom Province of Iran that had significant relationships with habitat variables. The value of the selectivity index is shown and each variable is divided to five intervals.

Analysis of deviance (Poisson error distribution) showed statistically significant influences of habitat variables on species abundance in 6 species, although the significant habitat variables differed among species (Table 2). Elevation, overall plant cover, annual plant cover and perennial plant cover and soil water absorption were the most important habitat variables that influenced lizard abundance. The overall patterns indicate one or more of these habitat factors significantly influenced the lizard abundance among different plots. For instance the abundance of *O. elegans* was significantly related to the overall plant cover, while elevation and soil water absorption were the main factors that influenced the abundance of *T. agilis* (Table 2). As we predicted, different habitat variables can influence the abundance of different lizard species, habitat selectivity (Ivlev's index) also varied among species (Figure 5). By indexing the habitat selectivity for each species, we determined the species preference according to their related habitat variables. *Ophisops elegans* avoided habitats with low overall plant cover (Figure 5a). *Bunopus crassicauda* preferred habitats with low annual plant cover (Figure 5b). *Trapelus agilis* avoided high elevations and mountain habitats (Figure 5c), and soils with moderate and high-water absorptions (Figure 5d). *Eumeces schneiderii princeps* preferred moderate elevations (Figure 5e) and avoided soils with low and high percentages of clay (Figure 5f) and moderate soil water absorption (Figure 5g), but these preferences were not very clear. *Phrynocephalus scutellatus* preferred habitats with a moderate annual plant cover (Figure 5h). *Mesalina watsonana* seemed to prefer habitats with moderate elevations (Figure 5j) and moderate soil water absorption (Figure 5k), but we could not identify a clear preference in this species according to perennial plant cover (Figure 5l). *Phrynocephalus maculatus* strongly preferred Site 12, which was a saline desert with low elevation and no vegetation. In addition, we captured one specimen of *L. caucasia* in a mountain habitat (Plot 6). However, this species is a rock specialist and we were unable to place pitfall traps in rocky terrain. 

## Discussion

The relationship between elevation and species diversity is an important one in ecology, and elevation gradients are often associated with changes in habitat variables and community structure [6,11,30]. The average elevation in the Central Plateau of Iran is about 900 m, but elevation increases to near 3000 m in some areas, especially in the Qom Province. Our results support the view of Heatwole (1982), that the species diversity of lizards declines with increasing elevation, as is also the case in birds and mammals [7]. Lizards often show high species diversity at intermediate elevations [17,31-33], but lower species diversity with increasing elevation. However, McCain (2010) pointed out that temperature is the most important habitat factor that correlates with these trends in species diversity across elevations. In this study, the highest species diversity occurred at an intermediate elevation (1289 m) with a 25% overall plant cover. Above and below that elevation, species diversity decreased. Hence, our results from 12 plots at different elevation gradients support the concept that species diversity peaks at mid elevations (e.g., in our Plot 11). 

Although elevation has a strong influence on lizard species diversity, habitat heterogeneity can alter the direct impact of elevation on lizard species diversity. Tews et al. [34] indicate in their review that 85% of studies from 1960 to 2003 found positive correlations between habitat heterogeneity and species diversity. In this study, lizard communities showed different patterns when habitat variables changed rapidly at the same elevation. At two low-elevation plots, the species diversity was lower (2.4) in one plot (plot 12 at 831 m) than in the other (3.6 for plot 5 at 836 m). A lack of vegetation and a high percentage of salt and clay were associated with the reduction in species diversity in this case. 

The different impact of each interaction on species diversity is a result of species adaptations and physiognomic conditions of the study area. For instance plots between 1000 and 2000 m did not show a clear pattern of species diversity according to elevation alone, when we added other habitat factors such as vegetation cover and soil water absorption, the pattern became clear and showed the maximum species diversity in sites with low altitude, low soil water absorption and medium plant cover. One of the explanations of this change in species diversity is that environmental variables have a different value for each species, and they can be species-specific, as we have shown for 6 species in the second part of our results. 

Processes of habitat selection in species involve different factors. One important aspect of this process is habitat variability [35,36]. As we have shown, habitat variables affect species diversity. The process of habitat selection by each species in our study sites explains how different habitat variables affect species abundance in each plot. In other words, response of lizard species to different habitat factors and the degree of their response to habitat factors (-1, 0, 1) is one of the reason that overall species diversity is varied among sites, even if some of the habitat variables are almost equal. 

The present study was designed to determine the effect of habitat variables on lizard species diversity based on pitfall trapping in Qom Province in Central plateau of Iran. This study has found that generally elevation is one of the main environmental variables that changes species diversity. The second major finding was that extreme change in one habitat variable can override the effect of another habitat variable and influence species diversity, such as species diversity in plot 12 and plot 5. 

This information can help us with future surveys to understand how these habitat changes could affect lizard species abundance and predict future changes in their abundance. This has implications for conservation management that can help us to determine how lizard species abundance is affected by a change in environmental variables due to human activity or climate change. 
